# Adherence to the Traditional Mediterranean Diet and Human Milk Composition: Rationale, Design, and Subject Characteristics of the MEDIDIET Study

**DOI:** 10.3389/fped.2019.00066

**Published:** 2019-03-15

**Authors:** Guido E. Moro, Enrico Bertino, Francesca Bravi, Paola Tonetto, Alberto Gatta, Pasqua A. Quitadamo, Gugliemo Salvatori, Claudio Profeti, Paola Di Nicola, Adriano Decarli, Monica Ferraroni, Alessandra Tavani, Bernd Stahl, Frank Wiens

**Affiliations:** ^1^Italian Association of Human Milk Banks (AIBLUD), Milan, Italy; ^2^Neonatal Intensive Care Unit, Department of Public Health and Pediatrics, Università degli Studi di Torino, Turin, Italy; ^3^Laboratory of Medical Statistics, Biometry and Epidemiology G.A. Maccacaro, Department of Clinical Sciences and Community Health, Università degli Studi di Milano, Milan, Italy; ^4^TIN-Neonatology Unit, IRCCS Casa Sollievo della Sofferenza, San Giovanni Rotondo, Italy; ^5^Neonatal Intensive Care Unit, IRCCS Ospedale Pediatrico Bambino Gesù, Rome, Italy; ^6^Azienda Ospedaliero Universitaria Meyer di Firenze, Florence, Italy; ^7^Nutricia Research, Department of Human Milk Research and Analytical Science, Utrecht, Netherlands

**Keywords:** maternal diet, Mediterranean diet, breast milk composition, fatty acids, anti-oxidative, oxidative load, lipid peroxidation

## Abstract

**Introduction:** Knowledge about how a lactating woman's diet influences the composition of her breast milk is still very limited. In particular, no study has evaluated the role of adherence to the Mediterranean diet on human milk characteristics.

**Aim:** We carried out an observational study to investigate the influence of mother adherence to a Mediterranean diet on her breast milk composition.

**Methods:** Between 2012 and 2014, 300 healthy mothers, who exclusively breastfed their babies, were enrolled from five centers across Italy. During a visit to the hospital center 6 weeks after childbirth these women were asked to provide a sample of their freshly expressed breast milk and to answer a series of questions on personal characteristics and lifestyle factors. The application of a validated food frequency questionnaire allowed the collection of detailed dietary habits. Milk was collected and then stored until chemical analyses were performed. The study has been registered (Trial Registration: Dutch Trial register NTR3468). Descriptive analyses on baseline characteristics of mothers and babies were carried out on the participants, overall and stratified by center.

**Results:** The participants had a mean age of 33 years (SD = 4.06), and a pre-pregnancy BMI of 22.3 Kg/m^2^ (SD = 3.22). Forty-seven percent gave birth to their first child, 40% to the second 13% to the third or subsequent child. Babies had a mean birth weight of 3,324 g (DS = 389), and a mean length of 51 cm (SD = 1.94). Fifty-three percent were males.

**Conclusion:** The present work provides the general description and the characteristics of mothers and babies included in the MediDiet study.

## Introduction

Efforts to understand the relevance of variations in breast milk composition due to maternal diet for the healthy development of the infant have been few (1). Most of these studies focused on the role of some overt micronutrient deficiencies in breast milk (2, 3). Yet, until today, the question whether dietary factors that are independent of micronutrient deficiencies also modulate the benefits of maternal milk for the respective infants has been mostly ignored by researchers.

There probably exists a plethora of dietary styles largely capable of preventing the occurrence of micronutrient deficiencies in lactating mothers and their babies. Within a wide spectrum some dietary styles have been found to be generally healthier than others in the general population. For example, compared to the Western diet, the Mediterranean diet has several known positive health implications on adults (4–8). A major part of the health advantage conferred by the Mediterranean diet is thought to be due to its typical profile of fatty acids (“FAs”) combined with dietary anti-oxidative factors and low oxidative load of foods consumed (9–15). Upon consumption, this combination is instrumental to the achievement of a favorable molecular structure and function of plasma lipids and cellular and extracellular membranes in various tissues and secretions (16, 17).

Specifically, we presume that the breast milk of women adhering to the Mediterranean diet bears a typical FA profile of its lipids, relatively low levels of oxidative damage to its precious, but vulnerable ingredients, and a relatively high anti-oxidative capacity.

We introduce here the rationale, design, and subject characteristics of the study MEDIDIET.

## Methods and Design

### Study Design

Within the MEDIDIET study, 315 healthy mothers, each breastfeeding a healthy infant, were recruited between October 2012 and June 2014 in five hospital centers across Italy to provide information on their diet and donate a sample of their breast milk for biochemical analysis. The participating centers were in the cities of Turin, Florence, Rome, San Giovanni Rotondo (“SGR”), and Palermo. Eligible women were invited to voluntarily participate in the study during their routine peripartum examinations at each hospital center. Participants were asked to provide a sample of their freshly expressed breast milk and answer a series of questions on personal factors that we considered modulators of breast milk composition and milk oxidative status at expression, during an extra visit to the center no sooner than 5 weeks and not later than 7 weeks after childbirth.

Of the 315 recruited women, 300 fully matched the inclusion/exclusion criteria (110 women from Turin, 23 from Florence, 46 from Rome, 101 from SGR, and 20 from Palermo). Specifically, to be included, women had to be healthy (free of diabetes, autoimmune diseases, cardiovascular disease, renal disease, and hypertension and sero-negative for HBV/HCV/HIV), non-smokers and non-abusers of drugs and alcohol, with a body mass index (“BMI”) <35 Kg/m^2^, aged between 25 and 41 years, and had residency in Italy as well as parents both born in Italy. Mothers being on a restriction diet due to food allergies and vegan mothers were excluded. Further inclusion criteria referring to the respective infant were: born at term (at gestational age 37–42 weeks), with a birth weight of 2,500–4,500 g and a body length of 46–56 cm, and being exclusively breastfed during the entire period between birth and milk donation at 6 ± 1 weeks of infant age.

Fifteen participating women were *post-hoc* excluded from the study for different reasons: not providing the milk sample (*n* = 1) or dietary information (*n* = 1), having infants aged >7 weeks at milk donation (*n* = 2), a BMI >35 Kg/m^2^ (*n* = 3), or the presence of disease conditions including celiac disease (*n* = 1) and hypothyroidism (*n* = 7).

### Interviews

Participating women were interviewed during their extra visit to the centers by centrally trained interviewers using a structured questionnaire including information on socio-demographic characteristics, anthropometric variables, lifestyle factors, a personal medical history, dietary habits, as well as use of nutritional supplements and over-the-counter-drugs during lactation.

### Dietary Assessment

Maternal dietary intake was investigated regarding four relevant time periods: (a) the postpartum period between birth and the 6 ± 1 weeks when milk sample was collected, (b) the last 2 days before the provision of the milk sample, (c) the last dinner before, and (d) the last breakfast before milk donation. We investigated the diet during periods (a) and (b) using a food frequency questionnaire (“FFQ”) with proven validity and reproducibility (18, 19). It contained questions on 78 foods and beverages, as well as additional questions on types of fat and oil used. Food consumption during the last dinner and breakfast [periods (c) and (d)] was elicited through open questions. Answers to questions about average frequency of consumption of the various foods, food groups or complex recipes for questions on periods (a) and (b) were coded as 0.5 occurrences per week or days, respectively, if occasional, and as 0 occurrences, if never consumed. Answers to questions on fat intake pattern, as well as portion size, were used to modulate the composition of recipes.

The food intake will be transformed to estimates of macro- and micronutrient intakes, as well as total energy intake, using an Italian food composition database and other sources when needed (20, 21).

One main purpose of the use of the FFQ was to enable assessment of mothers' adherence to the Mediterranean diet during periods (a) and (b) using different recognized dietary scores (22).

### Milk Collection

For providing a sample of breast milk, mothers were asked to visit the hospital center in the morning between 09:00 a.m. and 12:00 noon (after breakfast and before lunch). Milk collection was performed directly before the administration of the questionnaire during the same visit. Mothers were instructed to wash their hands carefully with soap and water before expressing 30–50 ml of their milk (foremilk) from one breast within 10 min by breast pump (Lactina Symphony, MEDELA AG, Baar, Switzerland) into sterile hard plastic containers. The time between milk sampling and last breastfeeding was recorded. Whithin 3 minutes the expressed milk was stirred and aliquoted into smaller sterile 10 ml plastic containers, overlaid with nitrogen gas, to prevent post-sampling milk oxidation (23) and tightly closed. The milk sample was then transported in an ice chest within 20–25 min to a freezer for storage at −70°C until analysis.

### Processing of Milk Samples

To clarify the effects of maternal diet on breast milk FAs the complete breast milk profile of FAs with chain length from 4 to 24 carbon atoms will be analyzed in terms of both phospholipids (“PL”) contained in the membranous outer layer of milk fat globules (MFG) and total FAs, made up ~98% by triacylglycerol from the core of MFG (24). Human milk FAs will be profiled as methyl esters by capillary gas chromatography and flame ionization detection (“cGC-FID”) with and without preparative separation of PL by high performance liquid chromatography and evaporative light scattering detection (“HPLC-ELSD”) according to the method proposed by Beermann et al. (25).

F_2_-Isoprostanes, specifically 15-F_2t_-isoprostane (8-iso-PGF_2α_) and its metabolite 2,3-Dinor-5,6-dihydro-15-F_2t_-isoprostane, some of the most prominent F_2_-IsoPs (26), will be measured by liquid chromatography (“LC”)-mass spectrometry (“MS”) (27). We will also measure two aldehydes, which can be formed as secondary lipid oxidation products, namely malondialdehyde (“MDA”) and hexanal (“HEX”). Quantification of MDA will be done after release, extraction, and derivatization by ion-trap GC-MS/MS with the incorporation of an internal standard (28). Volatile HEX will be analyzed by headspace GC-FID using a dedicated system (Heracles II, AlphaMOS, Toulouse, France).

In addition, we will test milk samples for the occurrence of oxidative protein carbonylation. Qualitative detection of protein carbonyls will be done by Western blotting and enhanced chemiluminescence (“ECL”) following derivatization with 2,4-dinitrophenylhydrazine and separation by molecular weight using polyacrylamide gel electrophoresis in the presence of SDS (“SDS-PAGE”) (29).

We will use two methods for estimating anti-oxidative capacity of milk samples; both are standard methods in food and milk analysis (30, 31). The Folin-Ciocalteu reagent (“FCR”) method (32) is based on an electron transfer reaction; the oxygen radical absorbance capacity (“ORAC”) method is based on a hydrogen atom transfer reaction (33).

The description of milk measurements is reported in Table 1.

**Table 1:** Overview of milk measurements.

**MILK QUALITY PARAMETERS**	
Fatty acid profiling of milk	Concentrations of individual fatty acids (“FAs”) with medium to very long chain-length in total fat and phospholipids; cholesterol concentration
Lipid peroxidation	Concentrations of malondialdehyde (“MDA”), hexanal (“HEX”), and F_2_-Isoprostanes (“F_2_-IsoP”)
Protein oxidation	Presence (yes/no) of carbonylated protein species per molecular weight class
Anti-oxidative capacity	Application of Folin-Ciocalteu reagent (“FCR”) method, and oxygen radical absorbance capacity (“ORAC”; both hydrophilic and lipophilic ORAC)
Macronutrient composition	Protein, lipid, carbohydrate (“CHO”), and energy density

### Statistical Analyses

All the collected data were transferred into an electronic database and processed using the SAS Statistical Package (SAS 9.4).

Results given in the present manuscript are based on means and standard deviation (“SD”), and percentiles (median, 10° and 90°) when variables were continuous, or percentage if the variables were categorical.

To investigate whether there were differences across centers with regard to different anthropometric, demographic, or lifestyle factors expressed on a continuous scale an Analysis of Variance (“ANOVA”) was performed after rank normal scoring transformation to avoid non-normal distribution (34). When the variables were categorical, a chi-squared test was performed.

All the analyses were carried out stratified by centers, in order to assess the presence of significant differences in baseline characteristics of mothers and babies, which may have an influence on both adherence to the Mediterranean diet and breast milk composition.

We a priori assumed that more than 10% of the study participants would be qualified as adherers to the Mediterranean diet (35). Based on this assumption and using published information on value distributions (SD) from other human milk studies (36–38) we calculated the expected statistical power of our study for testing our hypotheses. With a power >80% and a two-sided α = 0.05, more than 250 participants allow to detect a difference of < 20% between means of adherers and non-adherers to the Mediterranean diet for at least three investigated milk parameters: MDA, ORAC, and FCR. The protocol of the study included a preliminary contact with the potential participants during the perinatal visit, including the signing of informed consent; an extra visit was planned no sooner than 5 weeks and not later than 7 weeks after childbirth including the interview, the collection of food data and the donation of milk. Being concerned of a rate of non-return of mothers between the perinatal and the extra visit greater of 20%, each center was asked to make an effort to enroll more participants than the necessary, and they gathered more consensus during the extra visit than expected. At the end of the study the number of participants was 20% higher than expected.

## Results

Table 2 shows the distribution of selected characteristics of the 300 included milk's donors, stratified by center. The participants had a mean age of 33 years (SD = 4.06), with a significant difference across centers (*p* = 0.0436). The mean pre-pregnancy BMI was 22.3 Kg/m^2^ (SD = 3.22) without significant differences across centers (*p* = 0.0728). One hundred and forty-one (47%) participants gave birth to their first child, 119 (40%) to the second child, 40 (13%) to the third or subsequent child. There were no statistically significant differences in participants' parity across centers (*p* = 0.3968).

**Table 2:** Characteristics of 300 mothers by center.

	**Turin (*****n =*** **110)**		**Florence (*****n =*** **23)**		**Rome (*****n =*** **46)**		**SGR (*****n =*** **101)**		**Palermo (*****n =*** **20)**		**Total (*****n =*** **300)**		
	**Mean (SD) P10-P90**		**Mean (SD) P10-P90**		**Mean (SD) P10-P90**		**Mean (SD) P10-P90**		**Mean (SD) P10-P90**		**Mean (SD) P10-P90**		***P value***
Age of mother (years)	34 (3.67)		33 (3.53)		34 (4.31)		32 (4.02)		33 (5.40)		33 (4.06)		0.0436
	29–39		29–38		28–40		26–37		25–39		28–38		
Pre-pregnancy BMI (kg/m^2^)	22.1 (2.55)		21.1 (3.26)		22.3 (3.55)		23.0 (3.66)		21.7 (2.82)		22.3 (3.22)		0.0728
	19.1–25.1		18.4–26.6		18.9–27.9		18.9–27.9		18.4–25.7		18.8–26.5		
	**No**.	**%**	**No**.	**%**	**No**.	**%**	**No**.	**%**	**No**.	**%**	**No**.	**%**	***χ**^**2**^*
**PARITY, INCLUDING THE CURRENT INFANT**													
1	60	54.55	9	39.13	20	43.48	42	41.58	10	50.00	141	47.00	0.3968
2	40	36.36	12	52.17	21	45.65	41	40.59	5	25.00	119	39.67	
≥3	10	9.09	2	8.70	5	10.87	18	17.82	5	25.00	40	13.33	
**MOTHER'S PROFESSION**^**a**^													
Housewife, student, unemployed	11	10.28	1	4.34	8	17.39	52	51.49	4	20.00	76	25.59	< 0.0001
Managers and intellectual professionals	44	41.12	5	21.74	18	39.13	13	12.87	4	20.00	84	28.28	
Technicians and clerical workers'	37	34.58	12	52.18	17	36.96	21	20.79	11	55.00	98	33.00	
Service and sale workers	11	10.28	5	21.74	2	4.35	12	11.88	1	5.00	31	10.44	
Agricultural, craft and trade workers, and machine operators	4	3.14	0	0	1	2.17	3	2.97	0	0	8	2.69	
**SMOKING STATUS**													
Never	66	60.00	13	56.52	28	60.87	63	62.38	12	60.00	182	60.67	0.0596
Ex-smokers	25	22.73	9	39.13	16	34.78	16	15.84	7	35.00	73	24.33	
Ex-smokers since pregnancy	19	17.27	1	4.35	2	4.43	22	21.78	1	5.00	45	15.00	
**SMOKE-FREE HOUSEHOLD**^**a**^													
No smoke	89	80.91	23	100.00	37	80.43	101	100.00	18	94.74	268	89.63	< 0.0001
Yes smoke	21	19.09	0	0.00	9	19.57	0	0.00	1	5.26	31	10.37	
**PHYSICAL LIFE/OCCUPATIONAL ACTIVITY**													
Sedentary	42	38.18	3	13.04	11	23.91	0	0	5	25.00	61	20.33	< 0.0001
Standing	24	21.82	10	43.48	7	15.22	2	1.98	15	75.00	58	19.33	
Medium or higher	44	40.00	10	43.48	28	60.87	99	98.02	0	0	181	60.33	
**LEISURE TIME PHYSICAL ACTIVITY DURING BREASTFEEDING**^**a**^													
< 2 h/week	100	92.59	4	17.39	44	97.78	101	100.00	18	90.00	267	89.90	< 0.0001
≥2 h/week	8	7.41	19	82.61	1	2.22	0	0	2	10.00	30	10.01	

*Italy, 2012–2014*.

a *The sum does not add up to the total because of some missing values*.

*BMI, body mass index; SD, Standard Deviation; SGR, San Giovanni Rotondo*.

The distribution of the mothers' profession significantly varied across the five study centers, possibly reflecting typical differences in education and social class between regions (*p* < 0.0001). Linked to these social differences may be the observed geographic differences in the level of occupational or daily life physical activity, which were statistically significant (*p* < 0.0001).

As concerns smoking status, most mothers (182, 61%) never smoked, 73 (24%) were ex-smokers, and 45 (15%) had stopped smoking at the beginning of the pregnancy. The majority of participants (268, 90%) lived in a smoke-free house, with some differences across centers (*p* < 0.0001).

Table 3 shows the distribution of selected characteristics of the respective infants of the included mothers, stratified by center. Most infants were born at a gestational age of 39–40 weeks (159, 53%), with some differences across centers (*p* < 0.0001). Mean birth weight and length were 3,324 g (SD = 389), and 51 cm (SD = 1.94). Mean infant age at milk donation was 44 days, with some differences across centers (*p* < 0.0001).

**Table 3:** Infants characteristics by center.

	**Turin (*****n =*** **110)**		**Florence (*****n =*** **23)**		**Rome (*****n =*** **46)**		**SGR (*****n =*** **101)**		**Palermo (*****n =*** **20)**		**Total (*****n =*** **300)**		
	**No**.	**%**	**No**.	**%**	**No**.	**%**	**No**.	**%**	**No**.	**%**	**No**.	**%**	***P-value***
**SEX**													
Males	53	48.18	12	52.17	19	41.30	48	47.52	9	45.00	141	47.00	0.9171
Females	57	51.82	11	47.83	27	58.70	53	52.48	11	55.00	159	53.00	
**GESTATIONAL AGE (WEEKS)**													
37–38	22	20.00	5	21.74	21	45.65	24	23.76	5	25.00	77	25.67	< 0.0001
39–40	67	60.91	8	34.78	18	39.13	54	53.47	12	60.00	159	53.00	
41–42	21	19.09	10	43.48	7	15.22	23	22.77	3	15.00	64	21.33	
**MODE OF BIRTH**													
Vaginal	86	78.18	20	86.96	21	45.65	82	81.19	0	0.00	209	69.67	< 0.0001
Cesarean section	24	21.82	3	13.04	21	45.65	19	18.81	0	0.00	67	22.33	
Not available	0	0.00	0	0.00	4	8.70	0	0.00	20	100.00	24	8.00	
	**Mean (SD) P10-P90**		**Mean (SD) P10-P90**		**Mean (SD) P10-P90**		**Mean (SD) P10-P90**		**Mean (SD) P10-P90**		**Mean (SD) P10-P90**		
Birth weight (g)	3354 (380)		3360 (471)		3185 (361)		3344 (377)		3345 (435)		3324 (389)		0.1239
	2840–3920		2650–4070		2750–3690		2870–3810		2850–3988		2825–3855		
Birth length (cm)	50 (1.69)		50 (2.48)		50 (1.74)		51 (1.87)		50 (2.50)		51 (1.94)		0.0002
	48–52		47–53		49–53		49–54		48–55		48–53		
Age of infant at milk donation (days)	43 (4.91)		41 (4.79)		42 (5.52)		47 (5.43)		40 (5.55)		44 (5.68)		< 0.0001
	36–49		35–48		37–50		39–52		32–47		36–51		

*Italy, 2012–2014. SD, Standard Deviation; SGR, San Giovanni Rotondo*.

The distribution of the use of nutritional supplements or over-the-counter drugs during breastfeeding, stratified by center is reported in Table 4. Most participants (155, 51.7%) used at least one of these products. Among supplements or over-the-counter drugs users, 135 used vitamins, 128 used minerals, and 49 used ω-3 fatty acids (such as DHA and EPA from fish oil). The use of galactogogues, substances to stimulate milk production, was common only in San Giovanni Rotondo.

**Table 4:** Use of nutritional supplements and over-the-counter drugs by mothers, by center.

	**Turin (*****n =*** **110)**		**Florence (*****n =*** **23)**		**Rome (*****n =*** **46)**		**SGR (*****n =*** **101)**		**Palermo (*****n =*** **20)**		**Total (*****n =*** **300)**	
	**No**.	**%**	**No**.	**%**	**No**.	**%**	**No**.	**%**	**No**.	**%**	**No**.	**%**
**USE OF NUTRITIONAL SUPPLEMENT OR OVER-THE-COUNTER-DRUGS**												
No	64	58.2	7	30.4	16	34.8	46	45.5	12	60.0	145	48.3
Yes	46	41.8	16	69.6	30	65.2	55	54.5	8	40.0	155	51.7
TYPE OF NUTRITIONAL SUPPLEMENT OR OVER-THE-COUNTER-DRUGS^*^												
Vitamins	36		14		29		50		6		135	
Minerals	33		12		26		51		6		128	
Probiotics	1		0		0		3		1		5	
Omega3	0		5		21		23		0		49	
Galactogogues	0		0		0		6		0		6	
Over-the-counter-drugs	8		1		2		4		0		15	

*Italy, 2012–2014. SGR, San Giovanni Rotondo*.

* *Sums do not correspond to the total number of subjects, because of many subjects consuming multiple types of supplements*.

## Discussion

The main strength of the study is its design, which is set up and statistically powered to test the anti-oxidative hypothesis. Adequate sampling of human milk and collection of dietary data is crucial for reduction of confounding factors in the hypothesized link between diet and anti-oxidative milk protection. Milk collection procedures, designed to minimize confounding factors included the overlaying of milk with nitrogen gas in the storage tubes and the short lag time until freezing of samples at low temperatures for storage. The second cornerstone of the study design is the use of detailed FFQ, which has been validated for its usefulness in measuring regular diet during extended periods by others (19). The nutritional period best defining human milk's oxidative load and anti-oxidative capacity at expression is currently unknown and difficult to predict. Relevant periods may differ between different oxidative/anti-oxidative parameters. Therefore, collecting dietary data referred to four different time periods before milk sampling is likely to allow us to determine the temporal patterns involved in the modulation of human milk composition by maternal diet.

There are also weaknesses in the set-up of our study. We did not collect follow-up data on the health status of infants, which may be influenced by milk parameters. Any down-stream health effects of observed milk differences on the breastfed infants, therefore, will remain unclear.

Despite these weaknesses, we expect the results of the study will allow us to support or reject the predicted milk composition responses to maternal diet. Further, dietary information was self-reported and based on participant's recall. However, we used a reproducible and valid FFQ (18, 19), and believe that participants had limited a priori knowledge on the role of Mediterranean diet on breast milk composition, thus any bias in the recall of food intake should be minimal.

We anticipate our results to be representative at least for the general population of Italian mothers. In fact, the participant women's mean age of 33 years (without significant differences across geographic area) is similar to the Italian national mean age of parturient mothers within the age range allowed in MEDIDIET and the mean age of parturient mothers in Italy (32.2 and 31.4 years, respectively 2016).

To our knowledge, this study is the first to consider the role of maternal adherence to the Mediterranean diet for provision of anti-oxidative protection to human milk compounds, which may be an aspect of public health relevance as maternal milk's oxidative status may put infants on a certain health trajectory. A few studies have been carried out in Italy, relating any aspect of maternal diet to breast milk composition (39–42). Alberti-Fidanza et al. reported moderate correlations between maternal intakes of vegetables, vitamin C and β-carotene, and breast milk total antioxidant capacity (39). Scopesi et al. found positive correlations between maternal intake of SFA, and MUFA and corresponding concentrations in transitional milk, and maternal intake of PUFA and corresponding concentration in mature milk (41). Valent et al. observed only a weak correlation between maternal consumption of fish and selenium concentration in breast milk (42). Boniglia et al. considered the role of maternal protein and energy intake on milk content of protein, nitrogen and free amino acids and did not find any association (40).

Despite the above mentioned weaknesses, MEDIDIET will provide original human milk data in conjunction with maternal dietary data that may become a model for similar studies on the public health relevance of maternal dietary patterns on breastfed infants.

## Ethics Statement

The study protocol was approved by a central ethics committee on behalf of the Italian Association of Human milk Banks (AIBLUD) and local ethics committees at three out of the five participating centers. The administration at Turin and Palermo were informed about the study prospect through the full set of study documents, but did not consider it necessary to seek another formal approval by their local ethics committees. The written informed consent of study participants and medical doctors in charge was obtained after a consent interview guided by a detailed informed consent form (“ICF”) and data confidentiality assured. The study was registered (Trial Registration: Dutch Trial Register NTR3468). No incentive was offered or received by participants.

## Author Contributions

GM, AG, EB, CP, GS, MF, AD, BS, and FW designed the study. GM, AG, EB, PT, PD, PQ, CP, GS, AT, FB, MF, AD, BS, and FW conducted the field work and coordinated the study. FB and MF performed the statistical analyses. FB, MF, and FW wrote the manuscript.

### Conflict of Interest Statement

BS and FW are employees of Nutricia Research. The funder played no role in the study design, the collection, analysis or interpretation of data, the writing of this paper or the decision to submit it for publication. The remianing authors declare that the research was conducted in the absence of any competing relationships, whether personal, professional, commercial, financial or otherwise, that could be construed as a potential conflict of interest.

## Abbreviations

FAs: fatty acids
PUFAs: polyunsaturated fatty acids
ROS: reactive oxygen species
SGR: San Giovanni Rotondo
ICF: informed consent form
MDA: malondialdehyde
HEX: hexanal
F2-IsoP: F2-Isoprostanes
FCR: Folin-Ciocalteu reagent
ORAC: oxygen radical absorbance capacity
CHO: carbohydrate
LC-PUFAs: long chain polyunsaturated fatty acids
EPA: eicosapentanoic acid
DHA: docohexaenoic acid
MFG: milk fat globuli
PL: phospholipids
cGC-FID: capillary gas chromatography and flame ionization detection
HPLC-ELSD: high performance liquid chromatography and evaporative light scattering detection
LC: liquid chromatography
MS: mass spectrometry
ECL: enhanced chemiluminescence
SDS-PAGE: polyacrylamide gel electrophoresis in the presence of SDS
FFQ: food frequency questionnaire
MDS: Mediterranean Dietary Score
SD: standard deviation
ANOVA: Analysis of Variance
BMI: body mass index.
